# The Developmental Toxicity of Haloperidol on Zebrafish (*Danio rerio*) Embryos

**DOI:** 10.3390/biomedicines13081794

**Published:** 2025-07-22

**Authors:** Maximos Leonardos, Charis Georgalis, Georgia Sergiou, Dimitrios Leonardos, Lampros Lakkas, George A. Alexiou

**Affiliations:** 1 Laboratory of Zoology, Biological Applications and Technology Department, University of Ioannina, 45110 Ioannina, Greece; m.leonardos@uoi.gr (M.L.); charisgeorgalis@gmail.com (C.G.); zoology@uoi.gr (G.S.); 2 Department of Physiology, Medical School, University of Ioannina, 45110 Ioannina, Greece; l.lakkas@uoi.gr; 3 Department of Hematology, University Hospital of Ioannina, 45110 Ioannina, Greece; d.leonardos@uoi.gr; 4 Neurosurgical Institute, University of Ioannina, 45110 Ioannina, Greece; 5 Department of Neurosurgery, University of Ioannina, 45110 Ioannina, Greece

**Keywords:** haloperidol, zebrafish, toxicity, antipsychotic, behavior

## Abstract

**Background/Objectives:** Haloperidol is a typical antipsychotic drug widely used for acute confusional state, psychotic disorders, agitation, delirium, and aggressive behavior. **Methods:** The toxicity of haloperidol was studied using zebrafish (ZF) embryos as a model organism. Dechorionated embryos were exposed to various concentrations of haloperidol (0.5–6.0 mg/L). The lethal dose concentration was estimated and was found to be 1.941 mg/L. **Results:** The impact of haloperidol was dose-dependent and significant from 0.25 mg/L. Haloperidol induced several deformities at sublethal doses, including abnormal somites, yolk sac edema, and skeletal deformities. Haloperidol significantly affected heart rate and blood flow and induced pericardial edema and hyperemia in a dose-dependent manner, suggesting its influence on heart development and function. Embryos exposed to haloperidol during their ontogenetic development had smaller body length and eye surface area than non-exposed ones in a dose-dependent manner. **Conclusions:** It was found that haloperidol significantly affects the behavior of the experimental organisms in terms of mobility, reflexes to stimuli, and adaptation to dark/light conditions.

## 1. Introduction

Haloperidol is a prototypical drug of the butyrophenone class. It is the commonly used antipsychotic drug in the therapy of patients suffering from acute and chronic schizophrenia [1]. The drug is also indicated for the control of tics and vocal utterances of Tourette’s disorder, both in children and adults [2]. Haloperidol is effective for the treatment of patients with severe behavior problems, such as explosive hyperexcitability, as well as for the short-term treatment of hyperactive children who show an excessive motor activity with accompanying conduct disorders. Some of the usual symptoms are impulsivity, difficulty in sustaining attention, aggressiveness, mood lability, and poor frustration tolerance.

Despite its clinical efficacy haloperidol is associated with a range of adverse effects, including extrapyramidal symptoms, tardive dyskinesia [3] sedation and possible cardiovascular complications [4] neurotoxicity and specifically abnormal movement [5] toxicity of neuronal cells via NMDA receptor complex [6] induce adverse effects, including addiction, and even sudden death [7,8]. However, antipsychotic medications are used as therapeutic strategies for other chronic psychiatric conditions such as anxiety, depression, and bipolar disorder during pregnancy [9]. Thus, concerns have also been raised regarding its potential teratogenicity and developmental toxicity, especially during pregnancy, where its impact on embryonic development remains poorly understood [10]. The investigation of such undesirable side effects is important for the widespread use of drugs. This investigation can be conducted by using vertebrate model organisms.

The investigation of haloperidol toxicity was carried out with the help of a widely used experimental animal in biomedical research [11,12], the zebrafish. Zebrafish is an experimental model organism suitable for drug discovery [13], toxicological [14], and developmental studies [15]. Intriguing features of zebrafish is the ability to lay a large number of transparent eggs (approximately 300 eggs per mature female), its external fertilization, the almost transparent embryos during embryonic and post-embryonic development [16] the high growth rate, especially at early life stages, the small size of its larvae (about 4 mm), easy and low-cost maintenance, short generation time [17] the permeability of the chorion [18]. The high degree of gene conservation between zebrafish and mammals and consequently, the great similarity between the human genome and that of zebrafish (about 70% of human genes have at least one obvious ortholog, in relation to 80% of human genes with mouse orthologs) [11,19,20] allowed the rapid translation of screen results to humans [18]. The zebrafish and humans share many similarities in bioprocesses and genomes. The zebrafish has emerged as an ideal animal model suitable for behavioral studies due to the capability to study its locomotor activity [21,22]. Finally, the use of zebrafish in research falls under the 3R principles (Replacement, Reduction, and Refinement) when it comes to research [23]. The above makes the zebrafish a valuable tool in biomedical research [24,25,26].

Given the genetic and physiological similarities between zebrafish and humans, studying zebrafish embryos during organogenesis can provide crucial insights into the potential developmental effects of haloperidol exposure during pregnancy. Understanding the link between developmental, physiological, and behavioral responses to haloperidol may offer predictive value for long-term neurobehavioral outcomes.

This study aimed to assess whether haloperidol, a widely used antipsychotic, induces developmental toxicity in zebrafish embryos. To achieve this, we employed a series of innovative toxicity assessment methods, including acute toxicity assays, morphological deformity evaluations, cardiac function analysis, and behavioral assessments. By investigating the interplay between morphological, functional, and behavioral responses to haloperidol in a vertebrate model, we sought to determine whether early exposure to this drug during critical periods of heart and neural development could predispose individuals to altered behavior, including potential vulnerability to addiction-related behaviors in adulthood. The effect of the first-generation antipsychotic medications has not been studied in depth, and there is still very limited evidence regarding the outcomes in humans due to the exposure to antipsychotic medication during development [10].

## 2. Materials and Methods

### 2.1. Zebrafish Housing and Husbandry

Adult zebrafish of the wild-type strain (AB) were maintained in a zebrafish housing system (Zebtec “Active Blue” Tecniplast), at 28 ± 1 °C, pH 6.5–7.5, conductivity 500 ± 50 μS cm^−1^ with a 14-h light/10-h dark photoperiod (lights on at 8:00 a.m.). Feeding of the fish was performed twice a day with zebrafish commercial feed (Zebrafeed, Sparos, Olhão, Portugal), following common practices. Sexually mature zebrafish were used as genitors. The afternoon before the spawning, males and females at a ratio of 3:2 were placed into a breeding tank (iSpawn-S, 13-L, Techniplast S.p.a, Buguggiate VA, Italy) and kept separated with a horizontal net-barrier (females above/males below). In the morning of the next day, the light was turned on and the barrier was removed. A plastic net on the bottom of the tank prevents adults from eating the eggs. After spawning, the eggs were collected and pooled into E3 medium embryo buffer solution [27].

### 2.2. Zebrafish Toxicity Testing

Haloperidol (C_21_H_23_ClFNO_2_) of concentration 5 mg/mL was used to prepare solutions of various concentrations by serial dilution of the starting compound. The collected zebrafish eggs were inspected, and the unfertilized eggs and those that showed developmental disorders were removed. One day after fertilization (24 hpf), the eggs were mechanically dechorionated for the toxicity assessment experiments. Details about the toxicity testing are given in the Supplementary Materials.

### 2.3. Lethal Dose (LD_50_) Determination

Toxicity assays (LD_50_ values) and confidence limits (LD_25_ and LD_75_) were calculated by counting the number of surviving embryos from the exposure to haloperidol concentrations and non-exposed groups during the period of 96 h. The experimental design followed OECD Fish Embryo Acute Aquatic Toxicity (FET) Test 236 [28,29] with minor modifications adapted to the haloperidol toxicity study. 

The lethal effect and of various haloperidol concentrations is determined by comparison to the non-exposed group to identify the LD_50_. Dechorionated zebrafish embryos were selected and exposed to haloperidol at concentrations of 0.25, 1.00, 2.00, 3.50, 4.00, 5.50, and 6.00 mg/L. During the exposure period, the haloperidol solutions were renewed daily.

Embryos with no heartbeat were assessed as dead. Mortality as well as deformities were estimated at 24, 48, 72, and 96 h by using a stereo microscope (Olympus SZX7 Stereo, Olympus KL300 LED light, Olympus, Tokyo, Japan). The embryo mortality percentage was calculated by comparing the number of dead embryos or larvae at the time of observation with the initial number of embryos at the start of exposure. All phenotypic scoring conducted after haloperidol exposure was based on subjective comparisons, except for heart rate and body length measurements. The median lethal dose (LD_50_) of the acute toxicity experiment was calculated by using the Probit analysis by IBM SPSS Statistics software (v. 29.0).

#### 2.3.1. Deformity Assessment

The larvae were inspected for deformities after exposure to haloperidol. The pictures and videos of the developing embryos were carefully examined, and five different types of deformities were selected (yolk sac edema, pericardial edema, spinal cord deformities, blood cell aggregations, lower jaw deformities).

The percentage of deformities was calculated as the ratio of embryos with malformations to the total number of live embryos at that haloperidol concentration and time point.

#### 2.3.2. Developmental Toxicity

The effect of haloperidol on development was studied by examining specific morphological (body length, eye surface) and functional (heart rate, extent of pericardial edema) parameters during embryonic development.

#### 2.3.3. Somatometric Measurements

Body length measurements as well as eye surface of embryos were studied under the effect of haloperidol concentration and hours post-exposure at each time interval and concentration of haloperidol to evaluate the effect on developmental ontogeny. During the haloperidol exposure up to 96 h, the zebrafish larvae were observed, photographed, and videotaped using a stereo microscope (Olympus SZX7 Stereo). The total length from the tip of the mouth to the tip of the caudal fin was considered as body length. The surface area of the eye was measured by using the Danioscope software (version 1.2, Noldus, Wageningen, The Netherlands). The body length and the eye surface area of at least 20 larvae were measured under each test condition (concentration and time interval), and the experiment was repeated thrice.

### 2.4. Assessment of Haloperidol on the Cardiac Development Toxicity

The heart development and the heart rate of zebrafish were studied in relation to haloperidol concentration and hours of exposure, to evaluate its effects on developmental ontogeny. Embryos were continuously monitored during haloperidol exposure up to 96 h, with observations documented through imaging and video recordings using a stereo microscope (Olympus SZX7 Stereo). Notably, pericardial edema was frequently observed, prompting quantitative analysis of the pericardial area using Danioscope software (version 1.2, Noldus, The Netherlands). The experiments were conducted in three replicates. Details about the heart rate testing are given in the Supplementary Materials. The General Linear Model analysis (GLM) was used to examine the effect of haloperidol concentration and time interval on heart rate function and edemas.

### 2.5. Behavioral Analyses

Motor function and the reflexes were studied in relation to haloperidol concentration (non-exposed and haloperidol-exposed larvae) by performing various behavioral trials. This is a common practice when testing the bioactivity of drugs. The touching motor response (TMR) was evaluated by performing a light touch stimulus test applied to the rostral head area by a glass capillary needle to determine the responsivity of larvae to this stimulus. Larvae lacking any response to a touch or phenotypically unable to swim were excluded from further behavioral assessment.

#### 2.5.1. Locomotor Activity

The larval swimming activity in terms of distance moved and velocity in relation to alternating light conditions (dark/light) was analyzed to evaluate basal locomotor activity (BLA). The Visual Motor Response (VMR) test was employed to examine the larvae’s ability to adapt to changes in light conditions (dark/light cycling) after exposure to various concentrations of haloperidol.

#### 2.5.2. Touch-Evoked Motor Response (TMR) and Vibrational Startle Response (VSR)

To assess the integrity of the sensory-motor system, the touch-evoked motor response (TMR) was examined by applying a light tactile stimulus to the rostral region of zebrafish larvae using a fine glass capillary. Both the non-exposed group and the group exposed to haloperidol concentration of 0.25 mg/L exhibited expected motor reactions, indicating normal sensory system development.

The vibrational startle response (VSR) was further assessed to investigate potential alterations in reflexive behavior. A mechanical tapping stimulus was applied, and the total distance traveled by each larva within 10 s post-stimulation was recorded. This measurement provided insight into both startle reactivity and baseline locomotor activity in non-exposed embryos in relation to haloperidol-exposed embryos.

### 2.6. Statistical Analyses

Statistical analyses and graphics were performed with the aim of SPSS statistical software v29 (IBM Corp., Chicago, IL, USA). The concentration at which 50% of exposed specimens died (LD_50_) was estimated using Regression Probit analysis (the chi-square test, Pearson goodness of fit test, and 95% confidence interval). The effect of the concentration of haloperidol on the dependent variables (heart rate, body length, eye surface, pericardial edema) was studied with the aim of GLM analysis (general linear model) using the concentration as an independent variable and time (hours of exposure) as a covariate.

To assess larval locomotor activity, both distances moved and velocity of movement were recorded using EthoVision XT tracking software (version 14; Noldus, Wageningen, The Netherlands). Variations in locomotion under alternating light and dark phases were analyzed using a General Linear Model (GLM), with haloperidol concentration as the independent variable and time of exposure as a covariate. The vibrational startle response (VSR) was also evaluated using the same tracking software. Statistical significance was defined as *p* < 0.001, with *p* < 0.05 considered marginally significant.

### 2.7. Ethics Statement

All experimental protocols in this study were approved by the Research Committee of the University of Ioannina and the Veterinary Department of the Region of Epirus, 11044-29/7/2022. The protocols were in accordance with the Guide for the Care and Use of Laboratory Animals [29]. Moreover, all experiments were conducted using zebrafish embryos no older than 6 days post-fertilization (dpf). Thus, ethical approval was not required, in accordance with regulations (Directive 86/609/EEC and EU Directive 2010/63/EU). These directives permit the use of zebrafish embryos for research purposes up to the onset of independent feeding, typically around 5–7 dpf.

## 3. Results

### 3.1. Lethal Concentration (LD_50_) Determination

The toxicity of haloperidol is induced in a dose-dependent manner. The mortality rate rises in accordance with the concentration increases (Figure S1 of the Supplementary Material). The lethal dose concentration (LD_50_) was 1.941 mg/L, while the LD_25_ and LD_75_ values were 1.171 mg/L and 3.217 mg/L, respectively.

### 3.2. Morphological and Functional Alterations

**Figure 1:**
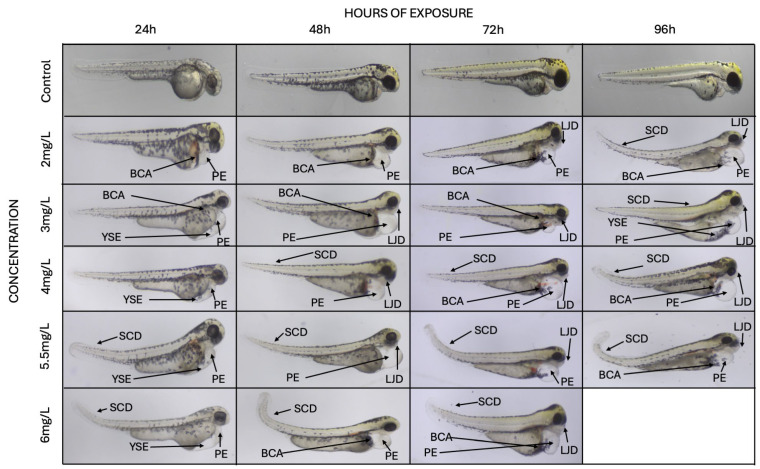
Morphological and anatomical alterations were observed in non-exposed and exposed zebrafish embryos to various concentrations of haloperidol. PE: Pericardial edema, YSE: Yolk sac edema, BCA: Blood cell aggregations, LJD: Lower jaw deformities, SCD: Spinal cord deformities.

**Figure 2:**
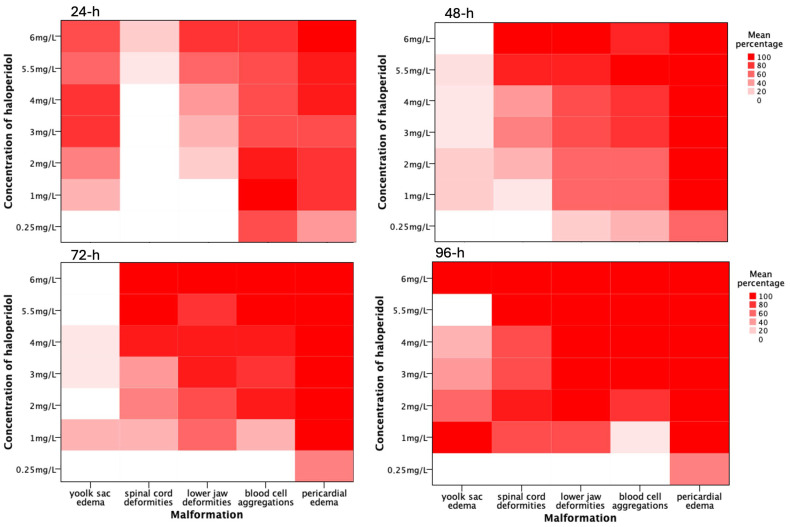
Heatmap of various malformations exposed to various concentrations of haloperidol up to 96 h of exposure.

Haloperidol-exposed zebrafish embryos exhibited dose-dependent morphological deformities, while no such deformities were observed in the non-exposed group during the study period (Figure 1). At sub-lethal concentrations (below the LD_50_), exposed embryos showed only minor developmental defects, including slight tail curvature, slight pericardial or yolk sac edemas. Conversely, exposure to concentrations exceeding the LD_50_ resulted in marked and progressively severe phenotypic and functional alterations, including spinal cord deformities (e.g., bending or twisting), pronounced pericardial and yolk sac edemas, and blood cell aggregation. The appearance and the intensity of malformations are dependent on the haloperidol concentration and hours of exposure (Figure 2).

#### 3.2.1. Pericardial Edema

Pericardial edemas began to appear even at 24 h of exposure and became more apparent until the 96 h. Notably, the myocardial contractility was affected (decreased) at higher concentrations. Of embryos exposed to haloperidol at 24 h, even at the lowest concentration (0.25 mg/L), 40% of specimens showed pericardial edemas (Figure 2). At the higher concentrations, more than 60% of the exposed specimens presented pericardial edemas. At 48 h, almost all embryos exposed to concentrations higher than 0.25 mg/L presented pericardial edemas (Figure 2).

#### 3.2.2. Blood Cell Aggregations

Of the exposed specimens, 70% showed few but obvious red blood cell aggregations at 24 h of exposure, even at a 0.25 mg/L haloperidol concentration. The appearance and the size of aggregations increased in a dose and time-dependent manner. At concentrations higher than 3 mg/L at 96 h, all the exposed specimens showed extended red blood cell aggregations.

#### 3.2.3. Skeletal Deformities

The occurrence of lower jaw and spinal cord deformities was found to be both dose and time-dependent. As haloperidol concentration increased and over time, the frequency and severity of these deformities also intensified (Figure 1 and Figure 2).

#### 3.2.4. Body Length

**Figure 3:**
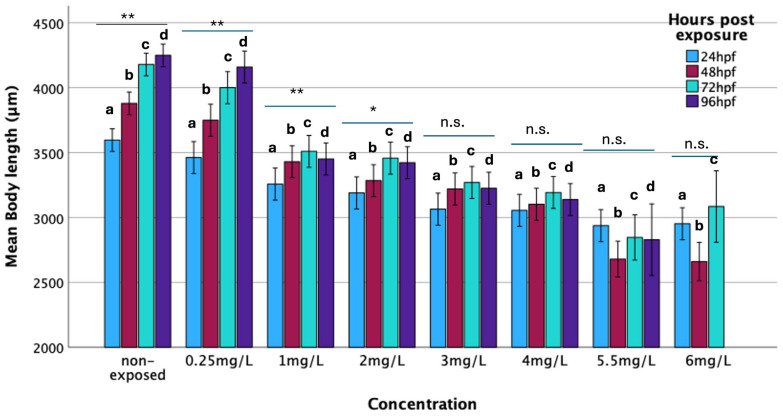
Mean body (μm) of zebrafish embryos’ body length exposed to various concentrations of haloperidol per time period (hours post-exposure). Significance: * *p* < 0.05, ** *p* < 0.001, n.s.: Non-significance. Comparison of mean body length in relation to concentration and hours post-exposure. (**a**) 24 h, F_1,7_ = 35.36, *p* < 0.001; (**b**) 48 h, F_1,7_ = 45.64, *p* < 0.001; (**c**) 72 h, F_1,7_ = 48.71, *p* < 0.001; (**d**) 96 h, F_1,6_ = 57.56, *p* < 0.001.

During the studied period (up to 96 h of exposure), the body length of the non-exposed embryos increased significantly over time, with a statistically significant difference observed across time points (F = 34,921.58; *p* < 0.001). A similar growth pattern was noted for embryos exposed to 0.25 mg/L of haloperidol (F = 58,954.05; *p* < 0.001) with no statistically significant difference compared to non-exposed specimens, suggesting a minimal effect of low-concentration haloperidol on body length. Conversely, embryos exposed to haloperidol concentrations of 1 mg/L and higher exhibited significantly shorter body lengths than both the non-exposed group and those exposed to 0.25 mg/L (Figure 3). No significant differences were observed between groups exposed to concentrations of 1 mg/L and above. At each time point, a progressive decrease in mean body length was observed with increasing haloperidol concentration. Generalized Linear Model (GLM) analysis confirmed that body length was significantly influenced by haloperidol concentration (F = 163.34; *p* < 0.001), time period (F = 25.31; *p* < 0.001), and their interaction (F = 5.60; *p* < 0.001).

#### 3.2.5. Eye Surface

**Figure 4:**
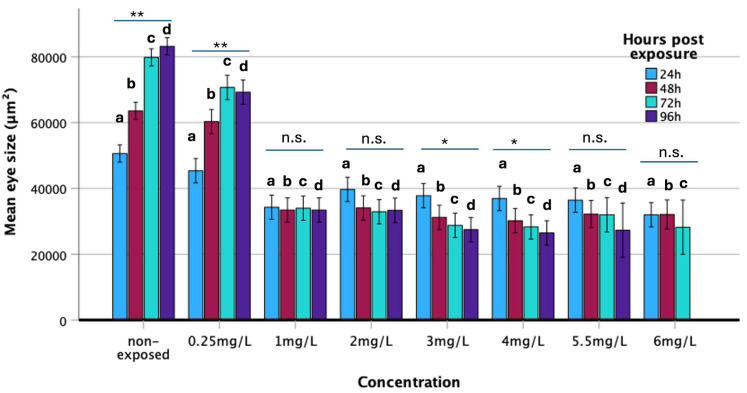
Mean eye size (μm^2^) of zebrafish embryos exposed to various concentrations of haloperidol per time period (hours of exposure). Significance: * *p* < 0.05, ** *p* < 0.001, n.s.: Non-significance. Comparison of mean eye size in relation to concentration and hours post-exposure. (**a**) 24 h, F_1,7_ = 26.32, *p* < 0.001; (**b**) 48 h, F_1,7_ = 88.51, *p* < 0.001; (**c**) 72 h, F_1,7_ = 168.01, *p* < 0.001; (**d**) 96 h, F_1,6_ = 112.54, *p* < 0.001.

The concentration of haloperidol had a statistically significant effect on eye surface area (F = 355.86; *p* < 0.001). Additionally, eye surface area was significantly influenced by time (F = 3.07; *p* = 0.028) and by the interaction between concentration and time (F = 23.47; *p* < 0.001) (Figure 4). In non-exposed specimens, eye surface area increased significantly over time, following a similar pattern in embryos exposed to 0.25 mg/L of haloperidol. However, at 72 and 96 h of exposure, the mean eye surface area in the 0.25 mg/L exposure group was significantly smaller than that of non-exposed specimens. Embryos exposed to concentrations of 1 mg/L or higher exhibited a markedly reduced eye surface area, with a progressive decrease observed as haloperidol concentration increased over time.

### 3.3. Heart Rate

**Figure 5:**
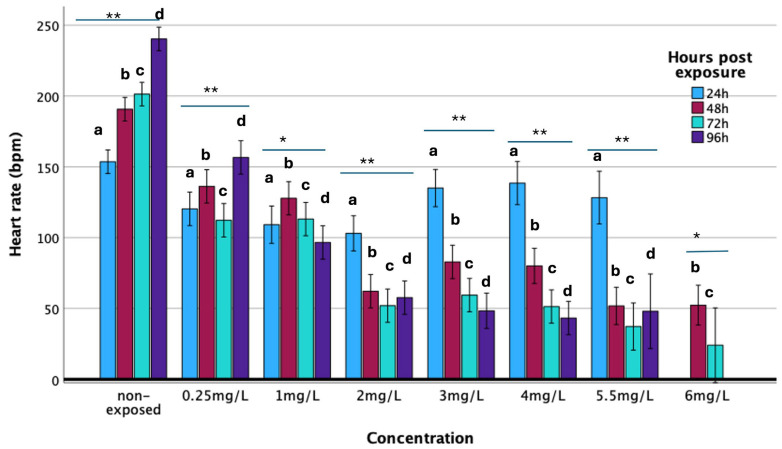
Heartbeat rate of zebrafish during embryonic development upon exposure to haloperidol at various concentrations at 24, 48, 72, and 96 h of exposure. Significance: * *p* < 0.05, ** *p* < 0.001. Comparison of mean eye size in relation to concentration and hours post-exposure. (**a**) 24 h, F_6,58_ = 12.32, *p* < 0.001; (**b**) 48 h, F_7,76_ = 124.20, *p* < 0.001; (**c**) 72 h, F_7,69_ = 75.42, *p* < 0.001; (**d**) 96 h, F_6,64_ = 256.77, *p* < 0.001.

The heart rate of developing embryos was expressed as the number of beats per minute (bpm) and was investigated in relation to exposure time (hours) and haloperidol concentration. The mean heart rate (stdev) of the non-exposed group was 153.60 (14.06), 190.65 (15.40), 201.30 (34.67), and 240.30 (10.25) (Ν = 20) at 24, 48, 72, and 96 h of exposure, respectively, exhibiting an increasing trend throughout zebrafish embryonic development (Figure 5). GLM analysis revealed a progressive increase in heart rate during embryonic development (F = 40.53; *p* < 0.001). Specimens exposed even at the lowest concentrations of haloperidol presented decreased heart rates, but also significant and severe arrhythmias. It has been found that haloperidol affects the heart rate by causing severe bradycardias. Increasing haloperidol concentration, the heart rate significantly reduced (F = 304.47; *p* < 0.001), while the combined effect of exposure duration and haloperidol concentration was also statistically significant (F = 28.69; *p* < 0.01).

### 3.4. Behavioral Analysis

#### Larval Activity

Haloperidol exposure induced concentration-dependent effects in zebrafish larval locomotor behavior, specifically in mean distance moved and velocity of movement, compared to the non-exposed group.

**Figure 6:**
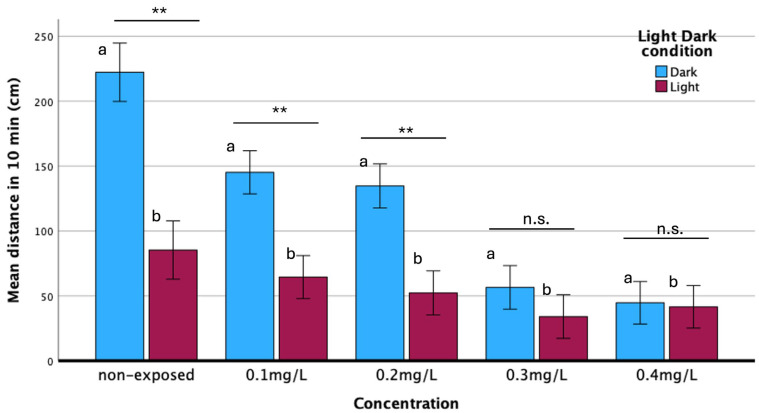
Mean distance moved (cm) of zebrafish embryos in 10 min, exposed to various haloperidol concentrations under dark/light conditions for 10 min. Significance: ** *p* < 0.001, n.s.: Non-significance. Comparison of mean eye size in relation to concentration and hours post-exposure. (**a**) Dark condition, F_4,386_ = 55.72, *p* < 0.001; (**b**) light condition, F_4,386_ = 4.16, *p* < 0.003.

The non-exposed group exhibited greater distance movement during the dark phase than in the light phase (Figure 6). However, the haloperidol-exposed group displayed reduced locomotor activity under dark/light cycling conditions with a characteristic decrease in mean distance moved (hypolocomotion). GLM analysis confirmed the statistically significant effects of haloperidol concentration (F _(1,4)_ = 44.42; *p* < 0.001), light/dark phase (F _(1_._1)_ = 126.76; *p* < 0.001), and their interaction (F _(4,4)_ =44.43; *p* < 0.001) on locomotor activity. The reduction in distance moved was more pronounced in the dark phase than in light, indicating that locomotor activity of zebrafish larvae under dark conditions was affected more than in light (Figure 6). For non-exposed specimens and those exposed to concentrations up to 0.2 mg/L, the distance moved during the dark phase was statistically greater than that during the light phase. However, at concentrations greater than 0.2 mg/L, there was no statistically significant difference, while at a concentration of 0.4 mg/L, the distances moved were almost equal. These findings indicate that haloperidol exposure exhibited significant hypolocomotion under both dark and light conditions and likely disrupts the ability of larvae to adapt their activity to dark/light transitions.

**Figure 7:**
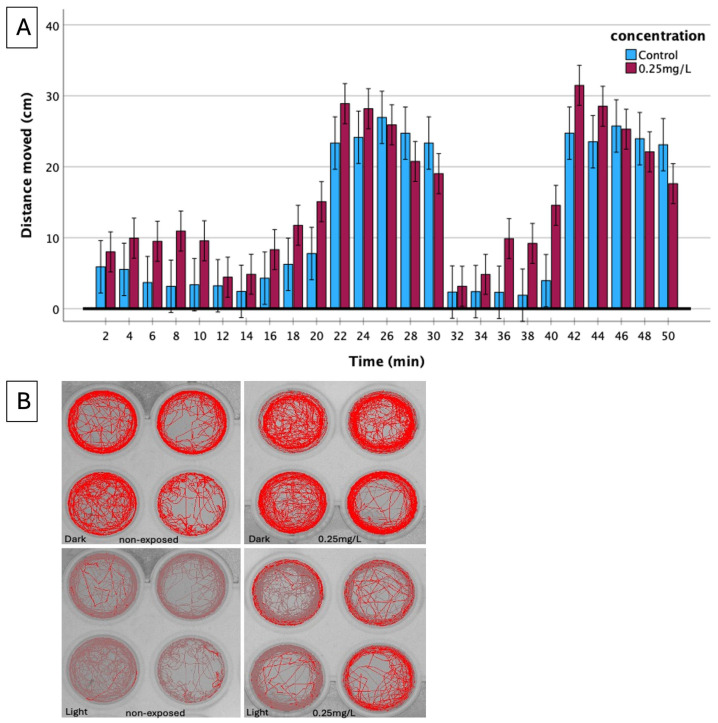
(**A**) The motor response of zebrafish larvae after 4 days of exposure is strongly impaired by haloperidol. Locomotor activity of non-exposed (*n* = 140) and exposed to 0.25 mg/L of haloperidol specimens (*n* = 240) was monitored in a 40-min period of light/dark cycling (10–20 min, light; 20–30 min, dark; 30–40 min, light and 40–50 min dark). The distance moved of exposed specimens was counted at intervals of 2 min and was compared with that of the non-exposed. (**B**) Track plot of zebrafish movement in the light and dark conditions, in relation to the concentration of haloperidol.

To further assess basal locomotor activity (BLA) in correlation to Visual motor response (VMR) under the effect of haloperidol, the mobility of zebrafish larvae was studied every 2 min time periods during the 10 min light/dark cycles. Initially, larvae underwent a 10-min acclimatization period in darkness, followed by alternating light/dark phases every 10 min. In the first five minutes of the light phase, specimens exposed to 0.25 mg/L showed greater mobility than the non-exposed ones (Figure 7A). They also showed greater mobility in the first 5 min of the dark phase, while in the next five minutes they showed less mobility than the non-exposed ones. In the light phase that followed, larvae exposed to 0.25 mg/L haloperidol showed statistically significantly greater mobility compared to the non-exposed ones. During the next 10 min of the dark phase, a similar trend was observed as in the previous dark phase.

Track plot study revealed that haloperidol-exposed larvae, even in lower concentration (0.25 mg/L), spend more time in the outer area of the well compared to non-exposed specimens (Figure 7B) (thigmotaxis).

A significant increase in the swimming distance was observed after applying a low-intensity stimulus to non-exposed and exposed larvae at 0.25 mg/L haloperidol.

**Figure 8:**
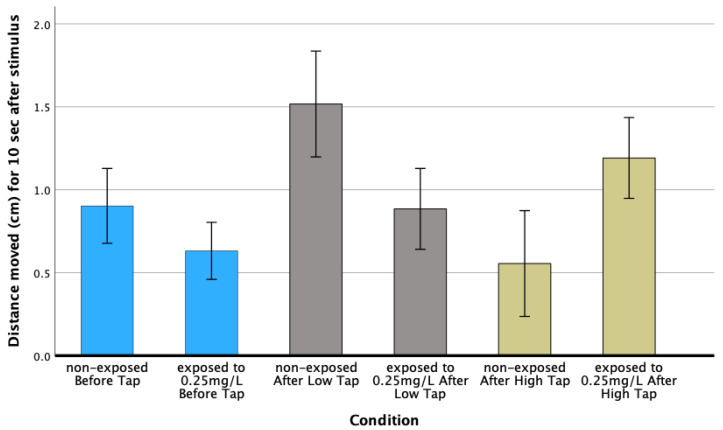
Distance moved (in cm) in 10 sec after stimulus delivery (Low and High) on zebrafish larvae, non-exposed and exposed to 0.25 mg/L haloperidol.

Moreover, after applying a higher-intensity stimulus (8×), the non-exposed larvae moved lower distances, while larvae exposed to 0.25 mg/L haloperidol moved about twice as far as before the stimulus (F = 13.71; *p* = 0.001) (Figure 8).

## 4. Discussion

Even though haloperidol has been used for decades, the knowledge about its effects on organisms and mechanisms of action, especially during ontogenetic development, still requires in-depth research. Thus, this study is focused on enhancing the understanding of how psychotropic drugs impact embryonic organogenesis, given the limited knowledge about the potential risks associated with antipsychotropic drug treatment. The general recommendations for haloperidol dose in pregnancy report that it should be the lowest effective for the shortest duration possible to manage symptoms while minimizing fetal exposure. Some experts recommend avoiding use during the first trimester, if possible, due to limited safety data, though no strong evidence links haloperidol to major congenital disabilities.

The susceptibility of zebrafish embryos to haloperidol was evaluated by evaluating the concentration that causes lethality in 50% of embryos exposed for 120 h post-fertilization, and 96 h of exposure was 1.941 mg/L. This is used in regulatory safety analyses to compare the toxicity across species.

Morphological malformations, mainly spine curvature and abnormalities of the lower jaw (Figure 1), were observed during this study. The abnormalities depended on the haloperidol concentration and on the exposure time. The frequency of occurrence increased depending on the concentration, as well as increasing over time (Figure 2).

Haloperidol [30], as well as other second-generation antipsychotics, can cross the placental tissue [10] and consequently affect the development of embryos. There is contradictory evidence on the increased risk of congenital malformations by antipsychotics [31]. Outcome data following exposure to typical antipsychotics do not show a significantly increased risk of major congenital malformations; however, outcomes may differ due to study design differences. It has been reported that children exposed to those drugs during the first trimester of development had an increased risk for congenital malformations [32]. Many of these results are confirmed by the present study. It was found that the dysmorphogenesis observed from the first 24 h of exposure and was dose and time-dependent (Figure 1 and Figure 2). However, there is a great need for in-depth investigation of malformations that are attributed to the haloperidol effect and may occur during organogenesis.

It was found that zebrafish embryos that had been exposed to various concentrations of haloperidol showed a reduction in their body length (Figure 3). This was dose- and time-dependent. The body length of the embryos decreased as the concentration of haloperidol increased, as well as the body length of the embryos also decreased over exposure time (Figure 3). It has also been reported that newborns whose mothers were taking antipsychotic medications had reduced birth weight [33].

Similar results were found when studying the surface of the eye. The analysis confirmed the toxic effect of haloperidol on the morphometry of the eye, as well as its neurotoxicity (Figure 4).

Malfunctions related to the cardiovascular system, mainly yolk sac edemas, pericardial edemas, and blood cell aggregations, were observed in a dose of haloperidol and time of exposure manner. Even at 24 h, the heart appears beating, although at a relatively irregular pace [16].

At 24 h of exposure (i.e., 48 h post-fertilization), the heart rate does not show statistically significant differences between the different concentrations. However, from the concentration of 0.25 mg/L, a significant decrease in heart rate is observed. As the concentration increases and over time, the heart rate decreases (Figure 5). Also, even from the lowest concentrations, it was found that pericardial edema occurred. The frequency of occurrence and the area of the pericardial edema were dose and time-of-exposure-dependent. Meaning that haloperidol has a significant effect on the development and function of the heart. There are several reports [4,8,34] of dysfunction, adverse effects, and sudden death in humans following exposure to haloperidol. Respectively, the surface of the pericardial area increased accordingly to haloperidol dose as well as the time of exposure (Figure S2 in Supplementary Data).

It was found that specimens exposed to concentrations of haloperidol greater than 1 mg/L, after 24 h from the beginning of exposure they presented a heart rate reduction of at least 50% of that of non-exposed specimens. These individuals subsequently died.

The findings of pericardial edema and bradycardia in zebrafish embryos exposed to haloperidol may result from either direct cardiotoxicity or indirect effects due to broader developmental disruption. While zebrafish offer a well-established model for assessing cardiogenesis and toxicological responses, it can be challenging to distinguish primary cardiac effects from those secondary to systemic developmental toxicity. For example, impaired cardiac function might stem from general delays in embryogenesis, altered neural regulation, or metabolic stress rather than direct action on the heart. However, evidence from human studies supports the notion that haloperidol has inherent cardiotoxic potential. Clinical data have linked haloperidol to QT interval prolongation, arrhythmias, bradycardia, and, in severe cases, sudden cardiac death [34,35]. These effects are primarily attributed to the drug’s ability to block cardiac potassium channels, particularly the hERG channel disrupting normal cardiac repolarization. Such findings suggest that the cardiovascular effects observed in zebrafish may reflect conserved mechanisms of direct toxicity. While we cannot exclude the contribution of general developmental toxicity, the presence of bradycardia and pericardial edema aligns with known cardiac risks associated with haloperidol in humans. Additional studies involving cardiac-specific gene expression, electrophysiological analysis, or rescue experiments could help to further clarify the underlying mechanisms. This study is useful in preliminary toxicological attempts to interpret sudden deaths after the administration of antipsychotic drugs on cardiovascular function and striated muscles [36,37,38] Our results will help reveal potential links between antipsychotics and unexpected physical dysfunctions that could trigger fatal accidents.

Both FGAs and SGAs can cross the placental tissue and, even though FGAs have been used in clinical practice since the 1950s [39] currently there is still very limited evidence regarding the outcomes in humans of the exposure to antipsychotic medication during development. In this regard, a systematic review and meta-analysis found an increased risk for major malformations in children exposed to both FGAs and SGAs [10]. The main organ affected was the heart, and there was also an association between antipsychotic gestational exposure and an increased risk of heart defects [33].

The behavioral study is a valuable tool to investigate the effect of haloperidol because the embryos from the 5 days post-fertilization respond to optical and acoustic stimuli [16]. The observed behavioral alterations in the exposed larvae were likely associated with cardiovascular dysfunction, disruptions in the muscular system, central nervous system (CNS) effects, and anxiogenic responses. Zebrafish larvae typically exhibit increased locomotor activity during dark phases and reduced movement under light, as quantified by the distance moved. This characteristic behavioral pattern was disrupted after haloperidol exposure (Figure 6). It was proved that a significant inverse relationship exists between haloperidol concentration and mean distance moved by zebrafish larvae, with locomotor activity declining as the concentration of haloperidol increased (Figure 6). This reduction was more pronounced during the dark phase than in the light. The observed hypoactivity at higher haloperidol concentrations could be attributed to morphological abnormalities that impair swimming ability. These findings are consistent with previous studies where similar decreases in movement were attributed to haloperidol-induced catalepsy [5]. Track plot analysis of zebrafish larvae (Figure 7B) suggests that haloperidol induces an anxiogenic effect under alternating dark and light conditions. Exposed to haloperidol larvae exhibited pronounced thigmotaxis, spending more time near the well’s periphery while avoiding the central zone regardless of lighting condition. This peripheral moving behavior is an indicator of anxiety in fish and was more pronounced following haloperidol exposure, and is reflected in the overall mobility, as expressed by the distance moved in both dark and light conditions (Figure 7B). Similar behaviors have been observed in zebrafish exposed to various anxiolytic drugs [36]. Also, zebrafish exposed to haloperidol moved near the bottom of the tank [5]; these results agree with those of the present study and confirm the significant effect on the behavior of the experimental animals.

A paradoxical agitating behavior of Zebrafish after exposure to dopamine antagonists has been previously described in the literature [21,38]. Specifically, [21] observed that D2-receptor antagonists, including haloperidol, induce an increase in waking activity during night periods. Interestingly, that behavior seems to be observed in a cluster of ether-a-go-go-gene-related (ERG) potassium channel-blocking compounds, including haloperidol [21]. Blockage of this channel is associated with prolongation of the QT interval in the heart rhythm and long-QT syndrome [40]. Hence, the initial agitation following haloperidol administration could be mediated via non-dopamine-dependent mechanisms. Despite the fact that the exact mechanism of agitation has not been well-described, previous studies have reported a dose-dependent effect of haloperidol in Zebrafish larvae [41,42] observed that low-doses of haloperidol, about 5 mΜ, increased the magnitude of motion index of zebrafish larvae at various time points, and found that an increase in locomotion parameters at low doses of haloperidol was more significant during periods of light exposure and at a drug concentration of 5.5 μΜ. However, that effect was blunt at higher concentrations. Despite that the mechanism of agitation have not been clarified, Mammalian studies have previously described that the initial agitation following low-doses of haloperidol administration could be mediated by blockage in the pre-synaptic D2 auto receptors, that cause an increase in the dopamine concentration in the synaptic cleft followed by blockage in the post-synaptic D2 receptors that finally results in suppression and in a biphasic time-dependent effect [43,44]. That time-dependent effect should be studied in future studies with longer observation intervals.

The increase in the distance moved after a low-intensity stimulus, both for non-exposed and exposed larvae at low concentrations (0.25 mg/L), is indicative of the body’s response to a stimulus. In the high-intensity stimulus, it was found that non-exposed larvae moved a shorter distance than before the stimulus, while those exposed to haloperidol traveled almost twice as far as before the stimulus, and greater than those exposed to lower intensity. The above are indicative of the effect of haloperidol on the reflexes of the larvae.

The shortened response time after an external mechanical stimulation with the tapping device indicates heightened sensitivity (Figure 8), which may be associated with haloperidol-induced stress and anxiety. From a behavioral perspective, the intense startle response observed suggests that haloperidol exposure enhances reactivity to sensory stimuli, consistent with a hyperactive startle behavior (Figure 8).

Zebrafish are widely recognized as a valuable model organism due to their remarkable regenerative capacity. They can regenerate a variety of tissues, including the brain, retina, spinal cord, heart, and others [45]. In this study, we observed that, behaviorally, the specimens generally exhibited recovery shortly after exposure. However, individuals exposed to concentrations exceeding the LD_50_ exhibited cardiac damage, and most failed to recover. Notably, exposure to a concentration of 6 mg/L resulted in mortality before the 96-h observation period was completed (Figure 5).

Further metabolomic analyses may provide insights into potential metabolites that are associated with increased neurodevelopmental toxicity. Measurements of oxidative stress markers (e.g., ROS) or apoptotic indicators (e.g., caspase activity) to strengthen claims of neurodevelopmental toxicity are planned.

## Data Availability

The data presented in this study are contained within the article and Supplementary Materials.

## Supplementary Materials

The following supporting information can be downloaded at https://www.mdpi.com/article/10.3390/biomedicines13081794/s1, Figure S1: Mortality pattern of zebrafish larvae exposed to different concentrations of haloperidol up to 96 hours of exposure; Figure S2: Pericardial area (mm^2^) of zebrafish embryos exposed to various concentrations of haloperidol per time period (hours of exposure).
