# Supplementary material for: The Developmental Toxicity of Haloperidol on Zebrafish (Danio rerio) Embryos
Source: Biomedicines. 2025 Jul 22;13(8):1794. doi: 10.3390/biomedicines13081794 (PMC12383424; doi:10.3390/biomedicines13081794)
Supplement: Supplementary file 1 [file biomedicines-13-01794-s001.zip › biomedicines-3697595-supplementary.pdf]

Supplementary Material

Experimental

Materials and Methods

Zebrafish toxicity testing

The dechorionated embryos were placed in 24-well culture plates (2 embryos per well, 1.5mL of solution per well, and E3 medium embryo buffer solution was used as solvent). Each experiment was performed in triplicate. Preliminary tests were performed to evaluate the range of 0%-100% mortality. The concentration range was found to vary from 0.25mg/L to 6.0 mg/L and E3 medium embryo buffer was used as control (non-exposed). In the current study, seven different concentrations of haloperidol were tested (0,25, 1.00, 2.00, 3.50, 4.00, 5.50 and 6.00mg/L). In total, 576 embryos were studied, of which 92 belonged to the non-exposed group. The total of the embryos of the non-exposed group were found to be alive, despite the manipulations during the dechorionation. Each experiment lasted about 96-h and began at 24 hpf, approximately at 26-somite point, according to Kimmel et al. Every 24-h, up to 96-h that the study lasted, the embryonic development status was checked, and the dead embryos were removed. During the experiments, toxicity, morphological abnormalities including yolk-sac edema, spinal curvature, tail deformity, uninflated swim bladder, and cardiac defects were recorded every 24 h. A stereoscope (Olympus BX43) equipped with a digital camera was used. High-resolution pictures were taken to be processed with the Image-Pro Plus software (Image-Pro Plus 11, Media Cybernetics). The recordings were performed at the same time (10 am) every day and lasted until the end of the experiment.

Each embryo was examined for apical endpoints such as: coagulation, lack of somite formation, non-detachment of the tail, and lack of heartbeat. Any positive result from one of the above four observations would make the embryo be considered as being dead. In relation to the heartbeat, particular care should be taken, since an irregular heartbeat should not be recorded as being lethal. Moreover, a visible heartbeat without any circulation in the aorta abdominals can be non-lethal. To record this endpoint, embryos showing no heartbeat should be observed under a minimum magnification of 80x for at least one minute. Absence of heartbeat is recorded after 24, 48, 72 and 96-h. Regarding the lack of somite formation, a normally developed embryo shows spontaneous movements (side-to-side contractions), which indicate somite formation. Non-formation of somites after 24-h might be due to a general delay in development. After 48-h, at the latest, the somites should have formed; if not, the embryos can be considered dead. In addition to the apical endpoints used for calculating the LD50, according to the guideline, the moment at which the embryos are hatched was recorded from 48-h. Any incidence of morphological and physiological abnormalities observed during the study in the results have also been recorded.
The data obtained from the apical endpoints were used to calculate: the survival percentage; the maximum concentration not causing mortality; the minimum concentration triggering 100% mortality; the mortality in the controls; the graph of the concentration-mortality curve at the end of the test. The median lethal concentration (LD_{50}) of the acute toxicity experiment was calculated from the data using the PROBIT function analyzed by IBM SPSS Statistics 29.0 software with 95% confidence limits.

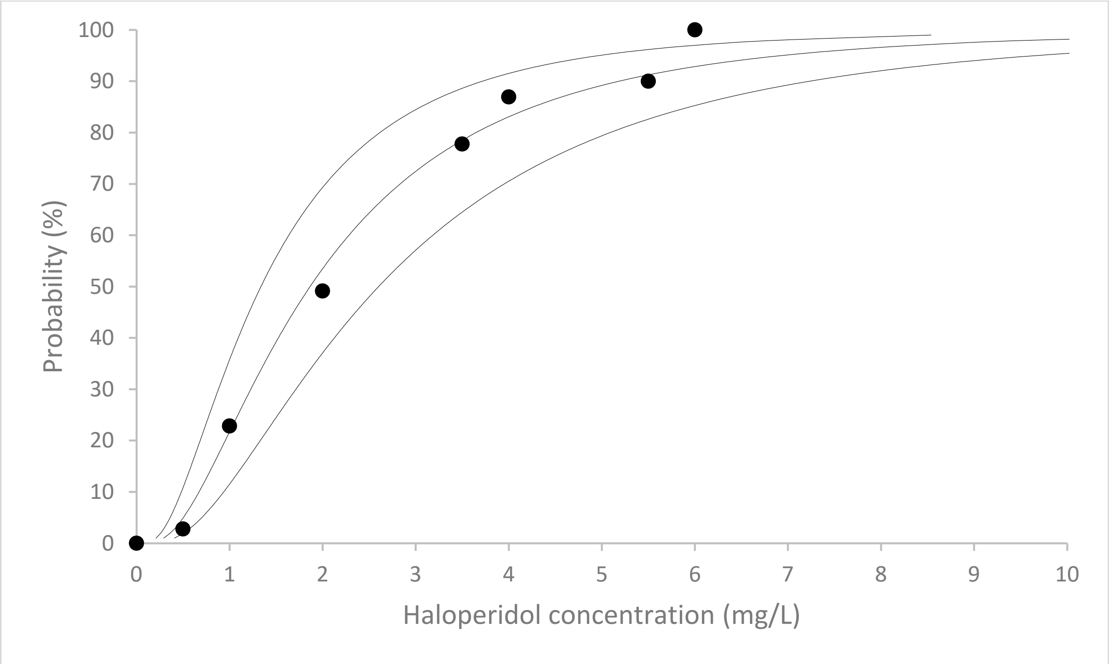

Figure S1. Mortality pattern of zebrafish larvae exposed to different concentrations of haloperidol up to 96 hours of exposure. The LC_{50} value was 1.941 mg/l, while LC_{25} was 1.171 mg/l and LD_{75} was 3.217 mg/l. Linear equation: y=3.073x+4.115 (R-sq=0.976).

Heart rate

The effect of haloperidol on heart rate as a function of its concentration and hours of exposure of embryos was studied, to evaluate the effect on developmental ontogeny. Forty at least embryos per concentration were separately analyzed under stereo microscope (Olympus SZX7 Stereo, Olympus KL300 LED light) and high-quality video recordings were obtained for 1 min using Basler MED Ace camera and Basler Microscopy software (Basler MED Ace 2.3 MP 164 color, Basler Microscopy Software V2.1). The video recording frame rate was set to 25fps. To eliminate any effect of temperature, the measurements were taken place in a temperature-controlled room (28 ^{o}C) and the video recordings were performed using transmitted cold lighting (LED). The acclimatization period was set at 20min before each recording. The heart rate of embryos, under the effect of various concentrations of haloperidol investigated on the first, second, third, and fourth day (24, 48, 72, and 96-hoe, respectively) after the starting of the exposure.

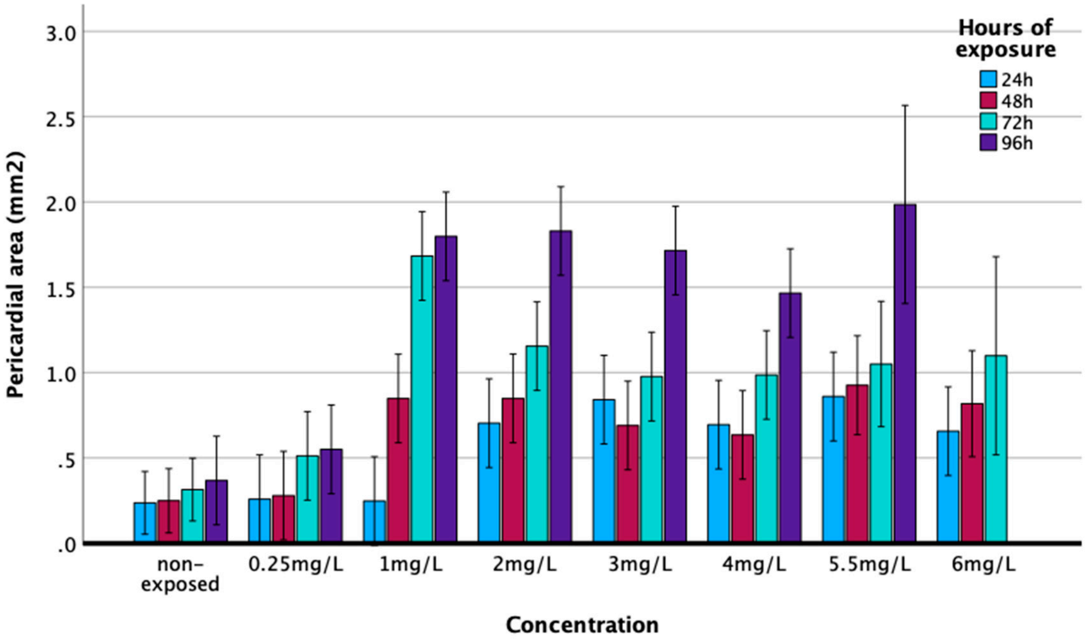

Figure S2. Pericardial area (mm^2) of zebrafish embryos exposed to various concentrations of haloperidol per time period (hours of exposure).
